# Measurement of circulating CD21^−^CD27^−^ B lymphocytes in SLE patients is associated with disease activity independently of conventional serological biomarkers

**DOI:** 10.1038/s41598-022-12775-4

**Published:** 2022-06-02

**Authors:** Alice Horisberger, Morgane Humbel, Natalia Fluder, Florence Bellanger, Craig Fenwick, Camillo Ribi, Denis Comte

**Affiliations:** Department of Medicine, Service of Immunology and Allergy, Lausanne University Hospital and University of Lausanne, 46, Rue du Bugnon, 1011 Lausanne, Switzerland

**Keywords:** Systemic lupus erythematosus, Autoimmunity

## Abstract

Determining disease activity in systemic lupus erythematosus (SLE) patients is challenging and limited by the lack of reliable biomarkers. Abnormally activated B cells play a key role in the pathogenesis of SLE, but their measure in clinical practice is currently not recommended. Here, we studied peripheral B cells to identify a valid biomarker. We analyzed peripheral B cells in a discovery cohort of 30 SLE patients compared to 30 healthy controls (HC) using mass cytometry and unsupervised clustering analysis. The relevant B cell populations were subsequently studied by flow cytometry in a validation cohort of 63 SLE patients, 28 autoimmune diseases controls and 39 HC. Our data show an increased frequency of B cell populations with activated phenotype in SLE compared to healthy and autoimmune diseases controls. These cells uniformly lacked the expression of CD21 and CD27. Measurement of CD21^−^CD27^−^ B cells in the blood identified patients with active disease and their frequency correlated with disease severity. Interestingly, we did not observe an increase in the frequency of CD21^−^CD27^−^ B cells in patients with clinically inactive disease but with elevated conventional biomarkers (anti-dsDNA and complement levels). Accordingly, measurement of CD21^−^CD27^−^ B cells represents a robust and easily accessible biomarker to assess the activity of the disease in SLE patients.

## Introduction

Systemic lupus erythematosus (SLE) is a chronic autoimmune disease characterized by a broad spectrum of clinical and immunological manifestations. While some SLE patients have a chronically active or monophasic course, most patients will present repeated flares interspersed with low clinical disease activity^1,2^. These flares may impact patients’ outcome due to the disease activity and increased exposure to immunosuppressive therapies^3^. Although disease activity inconsistently predicts future flares^4,5^, elevated disease activity scores indicate disease severity and predict the risk of organ damage^5^. Hence, determining disease activity in patients is crucial to refine therapy and limit the underlying inflammatory process leading to subsequent organ dysfunction. However, given the heterogeneity of SLE and the lack of consistent biomarkers, monitoring disease activity is challenging^6,7^. Identifying reliable, widely available, and non-invasive biomarkers remains an unmet need.

B cells play multiple key roles in SLE pathogenesis by producing pathological autoantibodies, secreting inflammatory cytokines, and presenting antigens^8–10^. For many years, they have been the target of treatments to control SLE disease activity. Belimumab, a treatment restraining B cell activation by blocking the B-lymphocyte Stimulator (BLyS), was the first therapy accepted for SLE treatment over the past 50 years. This successful phase III randomized control trial (RCT) in SLE patients, followed by a positive phase III RCT in patients with lupus nephritis, emphasized the pathological role of B cells in SLE disease^11,12^. Although initial trials on anti-B cell therapy involving rituximab—a type I anti-CD20 antibody leading to B cell depletion—failed to achieve primary endpoints in two separate studies^13,14^, improved trial design and a better understanding of SLE pathogenesis over the years have led to a recent positive trial outcome in a phase II RCT evaluating obinutuzumab, a humanized type II anti-CD20, in lupus nephritis^15^.

Examination of the altered distribution of peripheral blood B cells has been extensively researched for biomarkers. Despite the discrepancies in the classification of B cell subsets, studies have repeatedly reported an imbalance toward an increase in activated cells and antibody-secreting cells in SLE patients^16–23^. A decreased expression of CD21 characterizes activated B cells, and CD21^low/−^ B cell subsets are increased in SLE patients^17,24^. Distinct subsets of activated B cells were reported as an important source of antibody-secreting cells in SLE patients, similarly to conventional memory B cells. However, these cells lacked the typical memory marker CD27, lacked CXCR5 and expressed the myeloid marker CD11c^21,22^. In addition, upon stimulation, these atypical and activated B cells from SLE patients differentiated into plasmablasts and produced more auto-antibodies than the plasmablasts differentiated from classical memory B lymphocytes^21^. These distinct activated B cells were also identified in kidneys from patients with lupus nephritis, suggesting a direct pathological role of activated B cells in SLE^21,25^.

Disease activity has often been associated with these unbalanced profiles, making B-cell immunophenotyping a promising tool for evaluating future patients. However, at present, the measurement of B cell subsets in the peripheral blood of SLE patients is not a standard tool used in routine management, and its utility remains debated^26,27^ because of the lack of consensus in the classification of B cells and difficulties in reconciling results of the studies^24^. Furthemore, although the clinical application of immunophenotyping is widely used in other diseases such as HIV, hematological malignancies, and primary immunodeficiency^28–30^, there are no standardized procedures to assess the distribution of peripheral blood cells in patients with SLE. Finally, an important aspect to consider in the quest for biomarkers is identifying widely available tools. The increasing use of cutting-edge technologies such as mass cytometry and omics studies has allowed the identification of detailed subsets of functionally relevant B cells. Still, their importance as biomarkers in clinical practice needs to be further evaluated. In this study, we aimed to explore peripheral B cell subsets in two separate cohorts of patients, included in the Swiss cohort of SLE patients, to identify a potential biomarker. First, we used high-dimensional mass cytometry to precisely identify the B cell subsets that characterize SLE disease activity in a discovery cohort. Then, we translated our results by measuring relevant B cell populations by flow cytometry using a limited set of markers, and compared SLE patients to other autoimmune diseases in a validation cohort. Flow cytometry analysis were perfomed on fresh blood in concordance with regular routine follow-up laboratories and a limited set of surface markers to be representative of a real-life setting. We identified and validated that circulating CD21^−^CD27^−^ B cells were increased in SLE patients compared to controls. This population was associated with disease activity, independent of conventional biomarkers, and with severe clinical manifestations.

## Results

### Characteristics of SLE patients

B cell phenotype was evaluated in a total of 93 SLE patients, included in a discovery cohort A (n = 30) and a validation cohort B (n = 63). Detailed characteristics of SLE patients are available in Table 1. The patients included in both cohorts were mostly Caucasian women. Of note, there were more Asian and African patients in cohort B and the disease duration was longer than in cohort A. At the time of the study, the number of patients with high disease activity scores (SELENA-SLEDAI > 10) were similar in both cohorts. However, there were more patients with severe clinical features in cohort B, such as renal manifestations, neurological manifestations and vasculitis.

**Table 1:** Detailed characteristics of SLE patients from cohort A and B.

	Cohort A (n = 30)	Cohort B (n = 63)
Age, mean (SD) years	43 (14)	45 (13)
Female sex, no. (%)	25 (83)	56 (88)
**Ethnic background, no. (%)**		
Caucasian	27 (90)	44 (69)
Africans	1 (3)	7 (11)
Asian	2 (7)	9 (14)
Other	0	3 (5)
Disease duration, mean (SD) months	86 (73)	142 (175)
**Disease activity according to SELENA-SLEDAI**		
Inactive (0–3 points), no. (%)	17 (57)	28 (44)
Moderate (4–10 points), no. (%)	8 (27)	25 (40)
Active (> 10 points), no. (%):	5 (17)	10 (16)
**SELENA-SLEDAI score, mean (SD) and specific items**	5 (6)	7 (9)
Fever, no. (%)	1 (3)	2 (3)
Mucocutaneous^a^, no. (%)	8 (27)	16 (25)
Arthritis, no. (%)	4 (13)	6 (10)
Myositis, no. (%)	0	2 (3)
Serositis^b^, no. (%)	0	2 (3)
Renal^c^, no. (%)	4 (13)	25 (39)
Vasculitis, no. (%)	0	2 (3)
Neurological^d^, no. (%)	0	5 (8)
Hematologic^e^, no. (%)	3 (10)	36 (57)
Serologic, no. (%)		
Low C3 and/or C4	13 (43)	18 (33)
Positive anti-dsDNA antibodies	16 (53)	30 (48)
Physician Global Assessment score, mean (SD)	0.8 (0.8)	0.9 (0.9)
**Treatments past month**^**f**^		
No treatment	7 (23)	7 (11)
Antimalarials only, no. (%)	8 (27)	17 (27)
Systemic glucocorticoids, no. (%)	12 (40)	33 (52)
Dose, mean mg/day	10	9
Immunosuppressant agent, no. (%)	10 (33)	29 (46)

^a^Included rash, alopecia or mucosal ulcers. ^b^Included pleurisy or pericarditis. ^c^Included urinary casts, hematuria, proteinuria or pyuria. ^d^Included seizure, psychosis, organic brain syndrome, visual disturbance, cranial nerve disorder, lupus headache or cerebro-vascular accident. ^e^Included thrombocytopenia or leukopenia. ^f^Dose of systemic glucocorticoids corresponds to prednisone or equivalent, and immunosuppressant agents included azathioprine, methotrexate, and mycophenolate mofetil used during the last 4 weeks.

### Unsupervised clustering of B cells in SLE patients reveals increased subsets of B cells characterized by a lack of expression of CD21 and CD27

**Figure 1:**
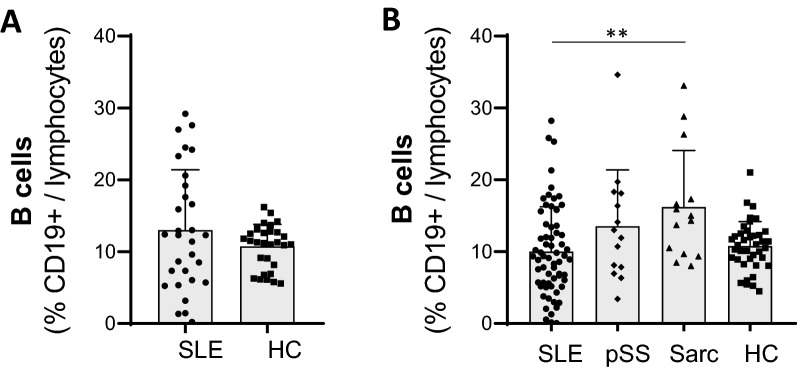
Frequency of B cells (CD19^+^) in systemic lupus erythematosus (SLE) patients compared to controls in two separate cohorts. (**A**) Frequency of B cells in SLE patients compared to healthy controls (HC): B cell analysis by mass cytometry followed by manual gating in 30 SLE and 30 HC (cohort A). (**B**) Frequency of B cells in SLE patients compared to other autoimmune diseases and HC: B cell analysis by flow cytometry in fresh blood of 63 SLE patients, 14 primary Sjögren’s syndrome, 14 sarcoidosis and 39 HC (cohort B). Bar plots represents mean ± SD. Statistical analysis were performed on log-transformed data (to obtain normal distributions) using a student-t-test (**A**) or a one-way ANOVA followed by Bonferroni’s correction (**B**), p value* < 0.05, p value ** < 0.01, p value *** < 0.001. Analylsis and figures were performed using GraphPad Prism version 8.0.0 for Windows (GraphPad Software, San Diego, California USA, www.graphpad.com).

**Figure 2:**
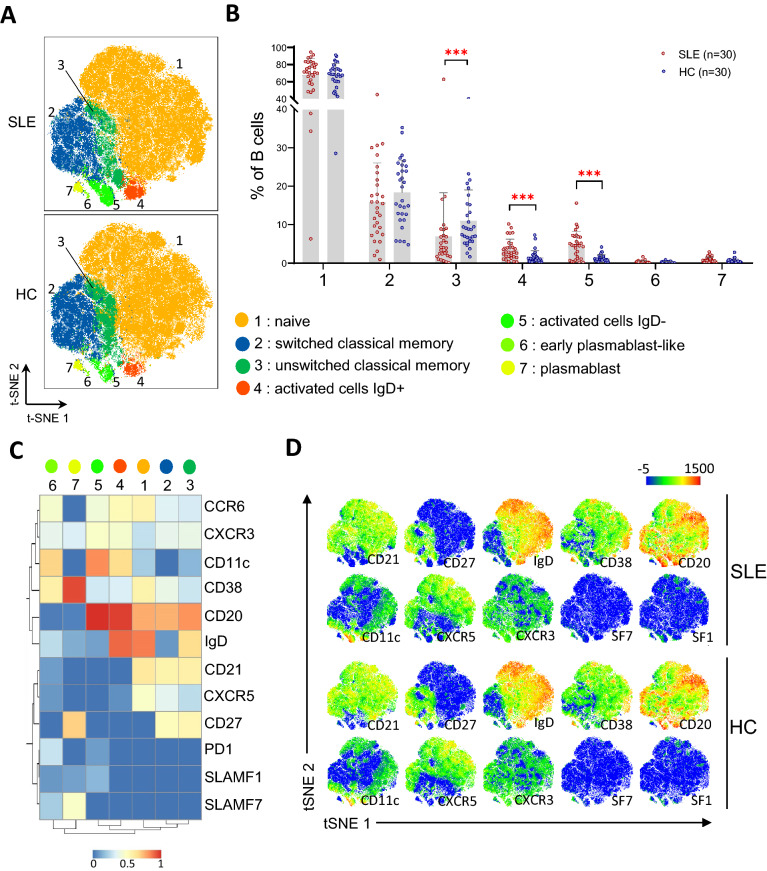
Analysis of B cells phenotype by mass cytometry in systemic lupus erythematosus (SLE) in comparison to healthy controls (HC) using unsupervised clustering analysis. (**A**) Merged t-SNE plots incorporating 120 k B cells from 30 SLE and 120 k B cells from 30 HC and showing B cell clusters identified by FlowSOM unsupervised clustering analysis (each color represents a different cluster). **(B)** Frequency of each B cell clusters in 30 SLE patients compared to 30 HC. Bar plots represents mean ± SD. Statistical analysis were performed on log-transformed data (to obtain normal distributions) using a student t-test followed by Bonferroni’s correction. ***p value < 0.001. (**C**) Heatmap showing B cell clusters and their scaled mean level of expression of each surface markers used for FlowSOM analysis. (**D**) tSNE plots showing the expression of surface markers (arcsin transformed) in SLE and HC. SF1 = SLAMF1, SF7 = SLAMF7. Analysis and figures were performed using GraphPad Prism version 8.0.0 for Windows (GraphPad Software, San Diego, California USA, www.graphpad.com) (**A**, **C**, **D**) and FlowJo™ Software version 10.7.1 (Becton, Dickinson and Company; 2019^59^) by exploiting the following FlowJo™ plugins: DownSample v3.3 and FlowSOM^60^ v2.9 (**B**).

In accordance with previous reports^17^, the frequency of B cells within lymphocytes was not significantly different between SLE and HC in both cohorts (Fig. 1). We first characterized the alteration of the SLE B cell compartment in discovery cohort A. To visualize B cells distribution according to their phenotype, we used a high-dimensionality reduction method (t-SNE) incorporating a total of 240,000 B cells (50% from SLE and 50% from HC) (Supplementary Fig. 1A). We identified seven B cell metaclusters using FlowSOM to perform unsupervised clustering (Fig. 2A). We found a significant increase in the abundance of metaclusters 4 and 5 and decrease in metacluster 3 in SLE compared to HC (Fig. 2B, Supplementary Fig. 1B). All samples had similar number of cells after downsampling, except for 5 SLE patients who had lower B cell counts (mean (range) B cells for these 5 SLE patients = 939 (375 to 1930)). Accordingly, we confirmed that our results remained consistent and significant after these samples were removed (Supplementary Fig. 1C). While metaclusters 4 and 5 differed in their expression of IgD, the two populations were characterized by lack of expression of CD21, CD27, and CXCR5, low CD38, and expression of CD11c and increased CD20 (Fig. 2C,D). The frequencies of metaclusters 4 and 5 were significantly correlated (r = 0.47, p = 0.0021). Based on our results and previous reports^21,22^, the cells from both metaclusters were identified as activated B cells.

In contrast, the frequency of metacluster 3, which presented a phenotype of classical unswitched memory B cells (USM), was reduced in SLE compared to HC (Fig. 2B,C). Other metaclusters were identified as naive (metacluster 1), classical switched memory (SM, metacluster 2) and plasmablasts (PB, metacluster 7) based on Fig. 2C,D and on the literature^24^. The frequencies of these metaclusters were not different between SLE and HC (Fig. 2B, Supplementary Fig. 1C). We identified metacluster 6 as potential early plasmablasts due to their close hierarchical clustering with plasmablasts, the negative expression of CD20 and their higher expression of CD38 and SLAMF7 in comparison to other non-PB cells. Due to the low number of cells in this cluster, metacluster 6 could also be the result of overclustering, which is generally recommended to capture small relevant cell clusters^31^. Nevertheless metacluster 6 frequency did not differ between the two groups.

### CD21^−^CD27^−^ B cells are increased in SLE patients compared to other autoimmune diseases

**Figure 3:**
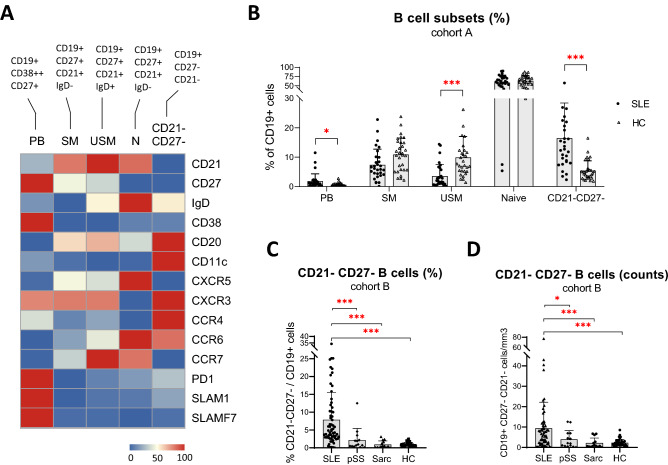
Analysis of B cell subsets using manual gating in systemic lupus erythematosus (SLE) compared to healthy and disease controls. (**A**) Heatmap showing mean levels of expression of surface markers in manually gated B cell subsets: naive (N), switched memory (SM), unswitched memory (USM), CD21^−^CD27^−^ B cell and plasmablast (PB). B cells were analyzed by mass cytometry in 30 HC and 30 SLE (cohort A). Data was normalized by rows. (**B**) Frequency of B cell subsets defined by manual gating using mass cytometry data in 30 SLE compared to 30 HC (cohort A). Bar plots represents mean ± SD. Statistical analysis were performed on log-transformed data (to obtain normal distributions), using a student-test to compare each population in SLE versus controls. (**C**, **D**) Frequency and absolute counts of CD21^−^CD27^−^ B cell, defined by manual gating of flow cytometry data, in 64 SLE patients compared to 14 primary Sjögren’s syndrome, 14 sarcoidosis and 39 HC (cohort B). Bar plots represents mean ± SD. Statistical analysis were performed on log-transformed data (to obtain normal distributions) using an ANOVA followed by Bonferroni’s correction, *p value < 0.05, **p value < 0.01, ***p value < 0.001. Analysis and figures were performed using GraphPad Prism version 8.0.0 for Windows (GraphPad Software, San Diego, California USA, www.graphpad.com).

Next, we performed manual gating of B cells in patients included in cohort A (Supplementary Fig. 2A, Supplementary Table 3). Because SLE disease is heterogeneous and the class-switching of activated B cells might be variable, we pooled manually all activated B cells as CD21^−^CD27^−^ based on our initial results. Frequencies of manually gated CD21^−^CD27^−^ B cells and metaclusters 4 + 5 significantly correlated (r = 0.43, p = 0.0027). In addition, activated B cells identified manually exhibited a cell surface phenotype (CD11c^+^CXCR3^+^CXCR5^−^CD38^−^IgD^+/−^) similar to that observed with unsupervised clustering (Fig. 3A, Supplementary Fig. 3).

Using manual gating, we confirmed the increase in CD21^−^CD27^−^ B cells and the decrease USM frequency in SLE patients compared to HC (Fig. 3B). In contrast to our initial results, we also observed a significant increase in total PB in SLE patients, as previously reported^19^.

Next, we performed a similar analysis in validation cohort B (Supplementary Fig. 2B, Supplementary Table 3). As expected, SLE patients had increased frequencies and absolute counts of CD21^−^CD27^−^ B cells compared to HC (Fig. 3C,D). Interestingly, SLE patients also had significantly more activated B cells than patients with other autoimmune diseases including pSS (Fig. 3C,D).

### CD21^−^CD27^−^ B cells correlate with clinical SLE disease activity

**Figure 4:**
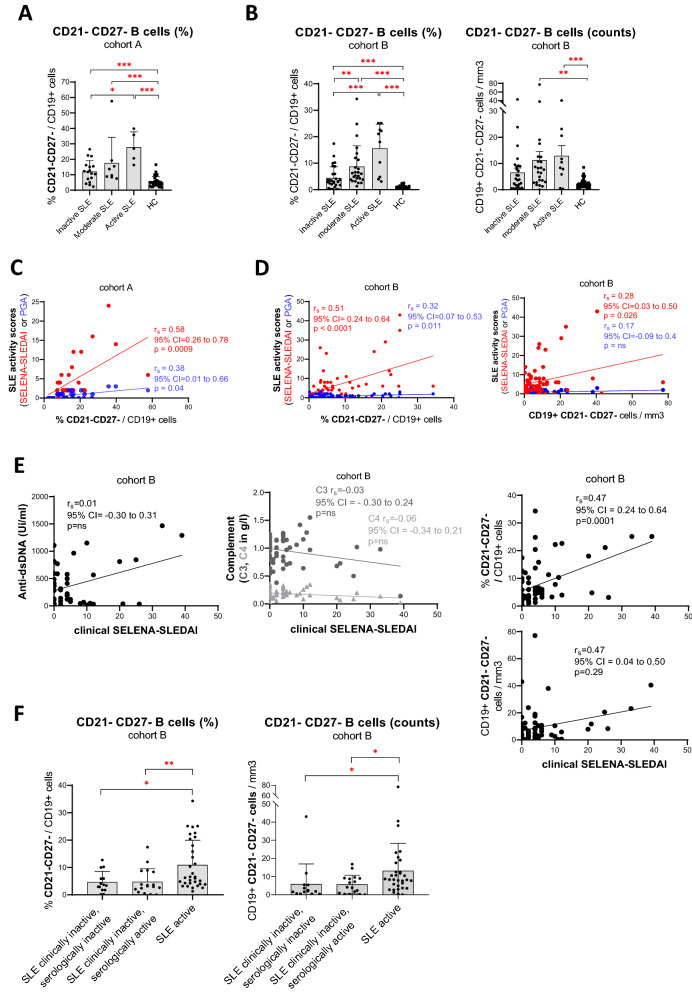
CD21^−^CD27^−^ B cell frequencies in systemic lupus erythematosus (SLE) patients according to disease activity parameters evaluated in two cohorts. B cells are analyzed by mass cytometry in 30 SLE and 30 HC in cohort A and by flow cytometry in 64 SLE and 39 HC in cohort B. (**A**, **B**) Frequencies of CD21^−^CD27^−^ B cell in SLE patients according to disease activity (cohort A (**A**): 17 inactive, 8 moderate, 5 active; cohort B (**B**): 28 inactive, 25 moderate, 10 active). (**C**, **D**) Correlation between disease activity scores (PGA and SELENA-SLEDAI) and the frequency of CD21^−^CD27^−^ B cell in SLE patients in cohort A (**C**) and cohort B (**D**). (**E**) Correlation between clinical SELENA-SLEDAI (SELENA-SLEDAI excluding anti-dsDNA and complement scores) and CD21^−^CD27^−^ B cell frequency, anti-dsDNA value and complement (C3 and C4) values in cohort B. (**F**) Frequencies of CD21^−^CD27^−^ B cell in SLE patients according to their clinical disease activity. Patients with disease activity limited to positive serology were classified as clinically inactive, serologically active. Bar plots in (**A**), (**B**), (**F**) represents mean ± SD and statistical analysis were performed on log-transformed data (to obtain normal distributions) using an ANOVA followed by Bonferroni’s correction. Statistical analysis for (**C**), (**D**) and (**E**) were performed using Spearman’s correlation. *p value < 0.05, **p value < 0.01, ***p value < 0.001. Analysis and figures were performed using GraphPad Prism version 8.0.0 for Windows (GraphPad Software, San Diego, California USA, www.graphpad.com).

By evaluating the relationship between CD21^−^CD27^−^ B cells and SLE disease activity, we observed a significant association with both activated B cell frequencies and absolute counts (Fig. 4A–D, Supplementary Fig. 5). Next, we used clinical SELENA-SLEDAI score, which excludes complement and anti-dsDNA from the score, as previously described^32^, to further evaluate the association between CD21^−^CD27^−^ B cells and disease severity in cohort B. We observed a significant correlation between CD21^−^CD27^−^ B cells (Fig. 4E) and clinical SELENA-SLEDAI. Of note, 9 (14%) SLE patients had high clinical SELENA-SLEDAI with a score > 10. These 9 patients had similar demographic characteristics compared to the general cohort B, apart from that all patients were females [female = 9 (100%), mean age = 46 yo and Caucasian = 6(67%)]. Of these 9 patients, 5 had neurological manifestations, 5 had renal manifestations and 2 had vasculitis, suggesting an association between CD21^−^CD27^−^ B cells and disease severity. In addition, 6 (67%) of these patient had a history of neuropsychiatic SLE and/or lupus nephritis (deterioration or new onset) involving either a hospitalization or an increased immunosuppressive therapy. Although our assessment of change in disease activity was limited by our initial study design and the limited access to restrospective data, our results may suggest an association between CD21^−^CD27^−^ B cells and severe flare^33^.

Of note, there was no correlation between clinical SELENA-SLEDAI and classical serum biomarkers (elevated anti-dsDNA antibodies and decreased complement C3, C4) (Fig. 4E). Additionally, patients with active disease had an increased frequency of CD21^−^CD27^−^ B cells compared with patients who showed no evidence of clinical activity but were serologically positive (increased anti-dsDNA and/or decreased complement, Fig. 4F). In fact, the frequency of CD21^−^CD27^−^ B cells was similarly low in clinically inactive SLE patients, regardless whether they were serologically active or not (Fig. 4F).

Next, to ensure the findings of the association between CD21^−^CD27^−^ B cells and disease activity were not due to confounding factors, we first tested whether each individual characteristics of SLE patients (demographic features, disease duration and treatments) was associated with the frequency of this cell population (Supplementary Table 4A). No association was found in cohort A. In cohort B, ethnic background (non-caucasian) and glucocorticoid dose were associated with a significant increase in CD21^−^CD27^−^ B cells. Detailed treatments received by each disease activity group of SLE patients are presented in Supplementary Table 4B. In a linear regression model, after adjusting for glucocorticoid dose and ethnic background in cohort B, CD21^−^CD27^−^ B cells remained significantly associated with disease activity, either as a continuous variable (SELENA-SLEDAI score : coefficient = 0.47, std error = 0.09, 96% CI = 0.28 to 0.66, p value < 0.001) or categorical variable (inactive, moderate and active SLE: coefficient = 5.21, std error = 1.16, 95% CI = 2.90 to 7.53, p value < 0.001).

Finally, as various definitions were used to define specific activated B cell subsets, we examined the importance of additional markers (CD11c, CD38, CXCR5, or IgD) for CD21^−^CD27^−^ B cells. These additional markers were chosen based on previously published reports examining B cell phenotype in SLE^22–24,34^. Compared to activated B cells defined as CD21^-^ CD27^-^ B cells, the addition of these surface receptors did not help identifying active disease (Supplementary Fig. 4).

These results show that the frequency of CD21^-^ CD27^-^ B cells is strongly associated with the clinically active SLE disease, independently from classical serological markers. In addition, activated B cells frequency is more accurate than anti-dsDNA antibodies elevation and complement components C3 and C4 decrease to help determine SLE activity and severity.

## Discussion

By using a limited set of common markers, we demonstrated an enrichment in CD19^+^CD27^−^CD21^−^ in the peripheral blood of SLE patients. B cells with a strong decrease in CD21 and CD27 expression were first identified by mass cytometry using an unsupervised analysis approach in a cohort of 30 SLE patients. Additionally, we confirmed our findings in a validation cohort of 63 SLE patients using flow cytometry, despite demographic and clinical disparities between both cohorts. Validation of a biomarker in a separate cohort of patients is an essential first step in the process of evaluating its clinical utility^35^. The increase in CD21^−^CD27^−^ B cells was associated with disease activity and severity of clinical manifestations. The correlation with disease activity was independent of treatment, including glucocorticoid dose. In contrast, patients with Sjögren’s syndrome and sarcoidosis did not display a significant increase in this cell population compared to healthy controls.

In our study, CD21^−^CD27^−^ B cells exhibited increased levels of CD11c and CD20 compared with other subsets. Additionally, they were CD38^low^, CXCR5^-^, and CXCR3^+^. Therefore, they resemble the previously reported activated B cells^21,24,36,37^. Accumulation of CD21^low^ B cells—expressing high levels of CD11c and the transcription factor Tbet—has been reported in many conditions, including common variable immunodeficiency, autoimmunity, and chronic infectious diseases^17,36–39^. Although the use of CD21 to classify distinct B cell subsets is still debated^24,38,40^, its downregulation identifies cells that exhibit an activation state^17,41^. Surprisingly, the use of the marker CD21^low^ alone on B cells (without CD27) was previously shown not to correlate with SLE disease activity^17^. In our study, increased B cells in SLE patients presented a phenotype of both CD21^−^ and CD27^−^ and this phenotype was significantly associated with disease activity. This suggests that identifying B cells using a strictly negative gate for CD21 (in contrast to CD21^low^) and adding CD27^−^ allows better association with disease activity. In contrast, the use of CD11c as an additional marker did not improve the ability to identify patients with active SLE. In line with our findings, the relationship between the frequency of CD11c^+^ B cells and disease activity is controversial^21,34^.

We observed that a proportion of CD21^−^CD27^−^ B cells expressed IgD, and that the others were switched (IgD^−^). From this point of view, Wang et al. reported that CD11c^hi^Tbet^+^ B cells were CD38^low^ and CD27^low^ and showed heterogeneous expression of IgD. They observed that both CD11c^hi^IgD + and CD11c^hi^IgD^−^ cells had similar transcriptome profiles and were able to differentiate into antibody-secreting cells^21,22^. Similarly, activated naïve (aNav) and double negative 2 (DN2) B are more numerous in SLE patients. These two cell populations, which are CD27^−^CD21^−^CD11c^+^CXCR5^−^, are thought to differentiate from the IgD + (aNav) to IgD^−^ (DN2) and terminally into antibody secreting cells independently from canonical germinal center reactions^22–24^. This non-germinal center pathway, also known as extrafollicular pathway, is suggested to be enhanced in SLE patients^22^. The differentiation of these cells is thought to be the result of a multistep process involving stimulation by TLR 7/9, interferon gamma and IL-21^42^. In our study, addition of the IgD marker to separate CD21^−^CD27^−^ B cells did not improve the association with disease activity in SLE. Because IgD expression could reflect different stages of the same pathological extrafollicular pathway, measurement of all CD21^−^CD27^−^ B cells could help identify SLE activity at various stages of the disease.

In this study, we observed a significant association between CD21^−^CD27^−^ B cells and disease activity, as measured by two distinct activity scores. The link between disease activity and CD21^−^CD27^−^ resembling B cells, such as CD11c^hi^Tbet^+^ or aNav/DN2 B cells, has been described previously. In contrast to previous studies, here, CD21^−^CD27^−^ B cells were not associated with the presence of anti-dsDNA autoantibodies^21,22,43^. In addition, we showed in our study that CD21^−^CD27^−^ B cells were not increased in patients who had only serological activity (increased anti-dsDNA and/or decreased complement) without any clinical evidence of activity. Classical serological markers have significant limitations in capturing SLE clinical activity and may remain positive in patients in remission^32,44^. Here we identified a biomarker that correlates with clinical disease activity independently of conventional serological biomarkers. The discrepancies between our findings and previous may be explained by distinct ethnic background, as the patients in our cohort were predominantly Caucasian. Furthermore, we did not observe a correlation between anti-dsDNA antibodies and clinical SELENA-SLEDAI. This was not surprising, as only a subset of SLE patients show an increase in anti-dsDNA over the course of their disease, while other may develop auto-antibodies against other nuclear antigens. In addition, the presence of anti-dsDNA antibodies was shown to be only inconsistently related to the activity of the disease, consistent with our results^7,44,45^. The increase in anti-dsDNA antibodies observed in patients with inactive disease could be the result of measurement of non-pathological anti-dsDNA of low avidity^46^. This may explain why we did not identify a correlation between anti-dsDNA and CD21^−^CD27^−^ B cell frequency. Overall, these findings underscore the need to identify new biomarkers. From this perspective, our data highlight the role of CD21^−^CD27^−^ B cells as an excellent biomarker of disease activity in SLE, especially in patients with inconsistent conventional biomarker results.

The increased population of CD21^−^CD27^−^ B cells observed in SLE patients was not correlated with age and disease duration. Other studies of resembling B cell populations in human SLE also found no association with age^21^. This is in contrast with the resembling mice cells called “age-associated B cells” (CD11c^+^CD11b^+^Tbet^+^ cells) that were originally described in the spleen of aged female mice^39^.

Our study showed that the critical expansion of CD21^−^CD27^−^ B cells is specific to SLE compared with pSS and Sarc controls. These data are consistent with those reported by Jenks et al., that show an expansion of DN2 primarily in SLE compared with other connective tissue diseases^22^. Interestingly, in pSS, only patients with pSS associated-lymphoproliferation have a significant increase in CD21^−/low^ B cells frequencies^47^. An altered distribution of circulating B cells has also been described in Sarc patients^48,49^. In our study, CD21^−^CD27^−^ B cells did not seem to be part of the abormaly distributed cells although the small sample size of patients with sarcoidosis may be a limiting factor.

This study is limited by its cross-sectional design. A longitudinal study would be needed to measure the sensitivity to change of CD21^−^CD27^−^ B cells over time as well as to assess their role as biomarkers predictive of flare severity. Interestingly, in a prospective study of 23 SLE patients treated with belimumab, the authors found a decrease in the frequency of CD11c^+^CD21^−^ B cells over time^50^. Secondly, this study included a majority of Caucasian patients. This aspect limits the interpretation of the results in patients of other ethnic background, and the utility of CD21^−^CD27^−^ B cells population as a biomarker of SLE disease severity in non-Caucasian ethnic groups should be further evaluated. Finally, we were unable to provide absolute cell counts for cohort A samples. This is because these samples were analyzed by mass cytometry and no counting beads were added to the samples.

Based on our findings, measuring CD21^−^CD27^−^ B cells in the peripheral blood is a promising biomarker of SLE disease activity. The fact that the results are consistent between the two distinct cohorts, which present demographic and clinical disparities, is an argument in favor of the usefulness of this biomarker. In addition, the analysis of cohort B was performed in real-life conditions (which includes immunophenotyping of fresh peripheral blood by flow cytometry on the day of the clinical appointement) and were not affected by treatment. In our opinion, our findings contribute to the field of SLE management by providing a valid and broadly accessible tool to improve the assessment of disease activity in clinical practice. Using a limited panel of three flow cytometry antibodies, these cells can be easily identified in blood samples from patients with SLE during follow-up visits.

## Material and methods

### Participant’s characteristics

We studied 93 adults included in the Swiss SLE Cohort Study (SSCS)^51,52^ and diagnosed with SLE according to the 1997 revised ACR criteria and/or the 2012 SLICC criteria^53,54^. Patients who received rituximab were excluded. Samples from two cohorts of SLE patients, separated in time, were analyzed by mass cytometry (cohort A) or flow cytometry (cohort B). In cohort A (discovery), we compared 30 SLE patients to 30 age-, sex- and ethnicity-matched healthy controls (HC; Supplementary Table 1A). Cohort B (validation) included 63 SLE patients and 39 age-matched HC as well as 14 patients with Sjogren's syndrome (pSS), meeting the 2002 American-European Classification Criteria^55^ and 14 patients with biopsy-proven sarcoidosis (Sarc; Supplementary Table 1B)^56^. pSS was selected as a disease control because both SLE and pSS are associated with B cell dysregulation and the two diseases exhibit overlapping clinical features^57^. Sarc was chosen as another disease control as it can accompany connective tissue disease and present with antinuclear antibodies. Altered distribution of circulating B cells has also been described in Sarc patients^48,49^, although it has primarily been considered a T-cell mediated disease.

Disease activity was assessed using the Safety of Estrogens in Lupus Erythematosus National Assessment modification (SELENA)-SLEDAI and the Physician's Global Assessment score (PGA)^33^. We categorized SLE patients into three groups of disease activity: inactive (SELENA-SLEDAI 0–3), moderate (SELENA-SLEDAI 4–10) and active (SELENA-SLEDAI > 10)^58^. Clinical SELENA-SLEDAI was used to evaluate disease activity, while excluding complement and anti-dsDNA from the score. All patients and controls were recruited at the Service of Immunology and Allergy of the Lausanne University Hospital and gave written informed consent. The study was approved by the Institutional Review Board (SwissEthics 2017-01434). All research was performed in accordance with the Declaration of Helsinki.

### Sample processing

For cohort A, peripheral blood mononuclear cells (PBMC) were enriched by density gradient centrifugation (FICOLL 400, Merck, Switzerland) and then cryopreserved in liquid nitrogen. For cohort B, we analyzed fresh blood by flow cytometry within a maximum delay of 24 h following sampling.

### Mass cytometry

Cryopreserved PBMC from SLE and matched HC were processed and analyzed as previously published^58^. Lists of mass cytometry antibodies are provided in the Supplementary Table 2 and were previously described^58^. To avoid batch effect, PBMCs of SLE and matched HC were barcoded, processed, and examined at the same time. Manual gating of FCS files was performed using FlowJo™ Software version 10.7.1 (Becton, Dickinson and Company; 2019^59^) on all cell populations from each samples. High dimensional data analysis was performed by exploiting the following FlowJo™ plugins: DownSample v3.3 and FlowSOM^60^ v2.9. Dimensionality reduction was realized using the Barnes-Hut implementation of t-stochastic neighboring embedding (t-SNE) algorithm on downsampled samples to obtain equal number of cells in SLE patients and HC. This is a common strategy used to analyze high dimensional data in mass cytometry analysis^61^. Unsupervised clustering analysis was performed using self-organizing map in combination with consensus clustering (FlowSOM) using 100 clusters.

### Flow cytometry

Fresh blood from SLE and controls were labeled, washed after red cell lysis (BulkLysis, Cytognos) and added to bead containing TruCount tubes (BD Biosciences) to obtain absolute counts. Lists of mass and flow cytometry antibodies are provided in the Supplementary Table 2 Cells were acquired on BD FACS Canto II (BD Biosciences).

### Statistics

Statistical analyses were performed using GraphPad Prism (version 8). We used parametric tests to compare the B cell populations in different groups of individuals as they were normally distributed after log-transformation. We used two-sided unpaired student t-tests to compare the cell frequencies or counts in two groups or one-way ANOVA to compare cells in more than two groups. For multiple comparison, we applied Bonferroni’s method for pairwise comparison of the means between groups. We used Pearson’s test for correlation between two log-transformed B cell subsets. As continuous clinical variable, such as SELENA-SLEDAI, remained skewed after log-transformation, we used Spearman’s rho test for correlation between clinical variable and CD21^−^CD27^−^ B cells. Linear regression model was used for multivariate analysis. p value < 0.05 was considered significant.

## Supplementary Information

Supplementary Figures.

Supplementary Tables.
