# Supplementary material for: Measurement of circulating CD21−CD27− B lymphocytes in SLE patients is associated with disease activity independently of conventional serological biomarkers
Source: Sci Rep. 2022 Jun 2;12:9189. doi: 10.1038/s41598-022-12775-4 (PMC9163192; doi:10.1038/s41598-022-12775-4)
Supplement: Supplementary file 1 — Supplementary Figures. [file 41598_2022_12775_MOESM1_ESM.pdf]

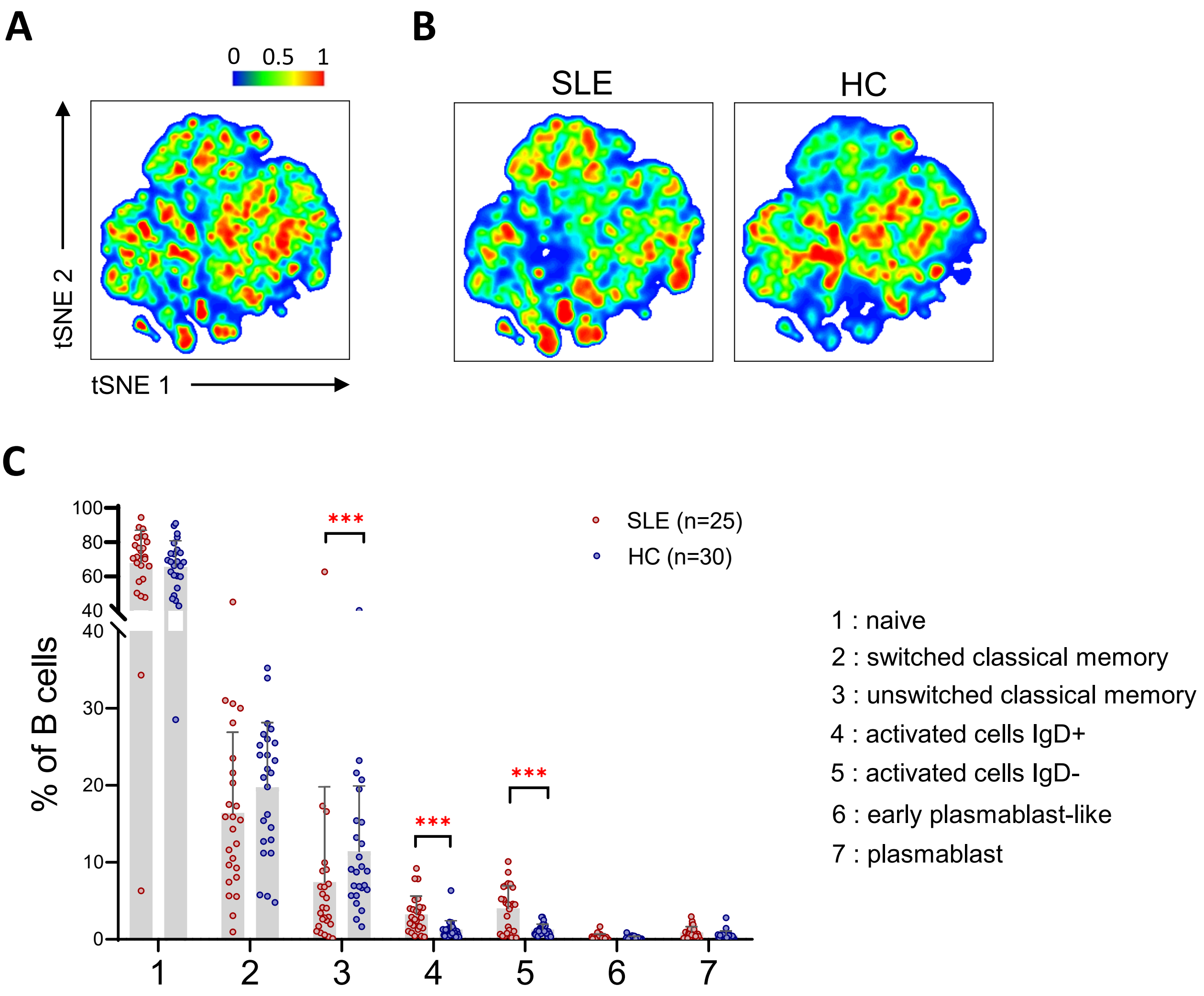

Supplementary figure 1. High dimensional analysis of B cells (A) Relative density of B cells mapped on the merged tSNE plot incorporating a total of 240,000 B cells, including 120,000 B cells from 30 healthy Controls (HC) and 120'000 B cells from 30 systemic lupus erythematosus (SLE) patients. **(B)** Relative density of B cells for each group mapped on the tSNE plots incorporating 120,000 B cells from SLE (left panel) and 120,000 B cells from Control (right panel). **(C)** Frequency of each B cell clusters in 25 SLE patients compared to 30 HC. Five SLE patients were excluded from this analysis because of low B-cell counts. Scatter bar plots represents mean ± SD. Statistical analysis were performed on log-transformed data (to obtain normal distributions) using a student t-test followed by Bonferroni's correction for multiple comparisons. *** p value < 0.001. Analysis and figures were performed using FlowJo™ Software version 10.7.1 (Becton, Dickinson and Company; 2019)(61) by exploiting the following FlowJo™ plugins: DownSample v3.3 (**A-B**) and GraphPad Prism version 8.0.0 for Windows (GraphPad Software, San Diego, California USA, www.graphpad.com) (**C**).

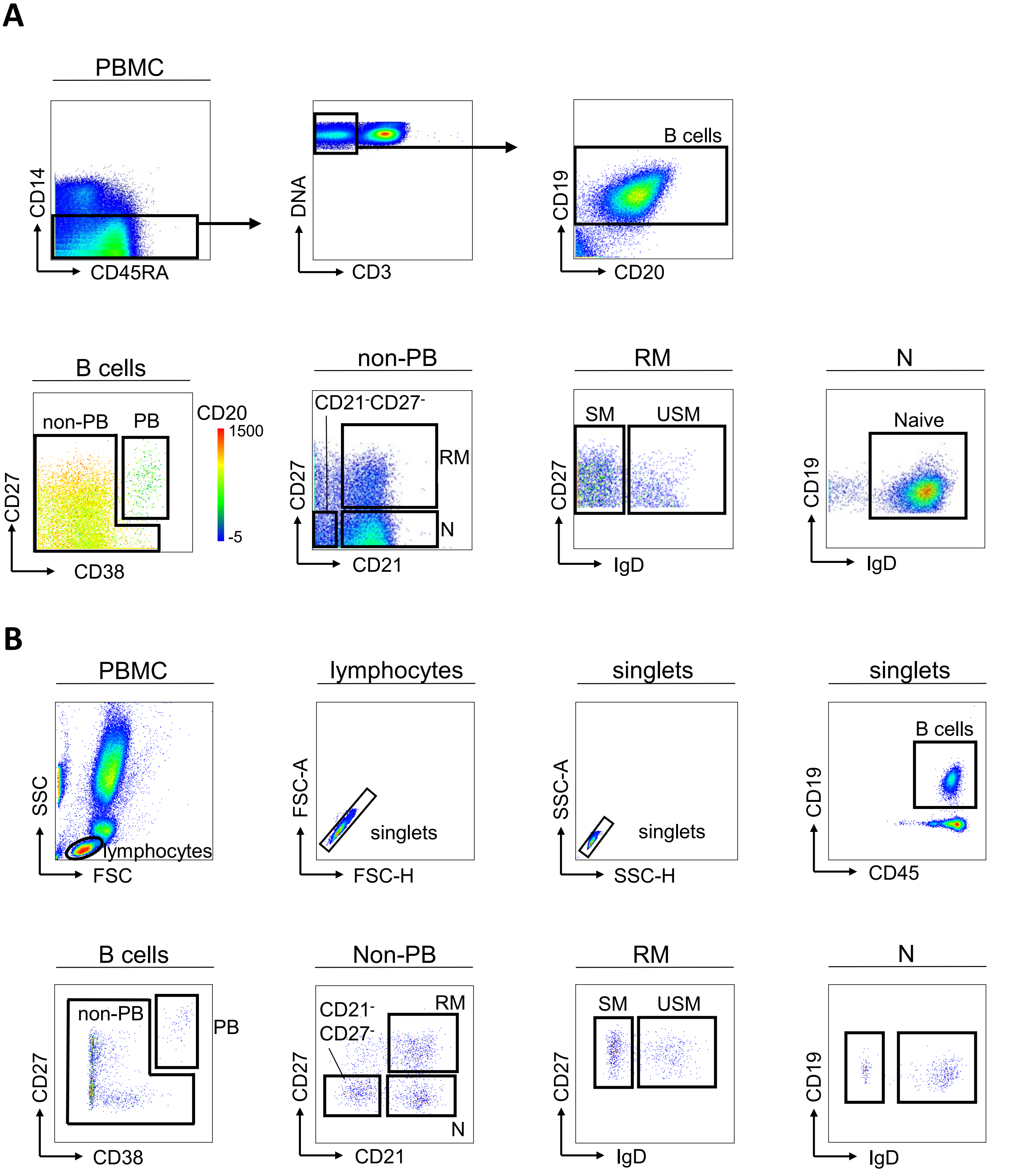

Supplementary figure 2. Manual gating strategy of B cell subsets analysis for mass cytometry data analysis **(A)** and flow cytometry data analysis **(B)**. Figures were performed using FlowJo™ Software version 10.7.1 (Becton, Dickinson and Company; 2019)(61).

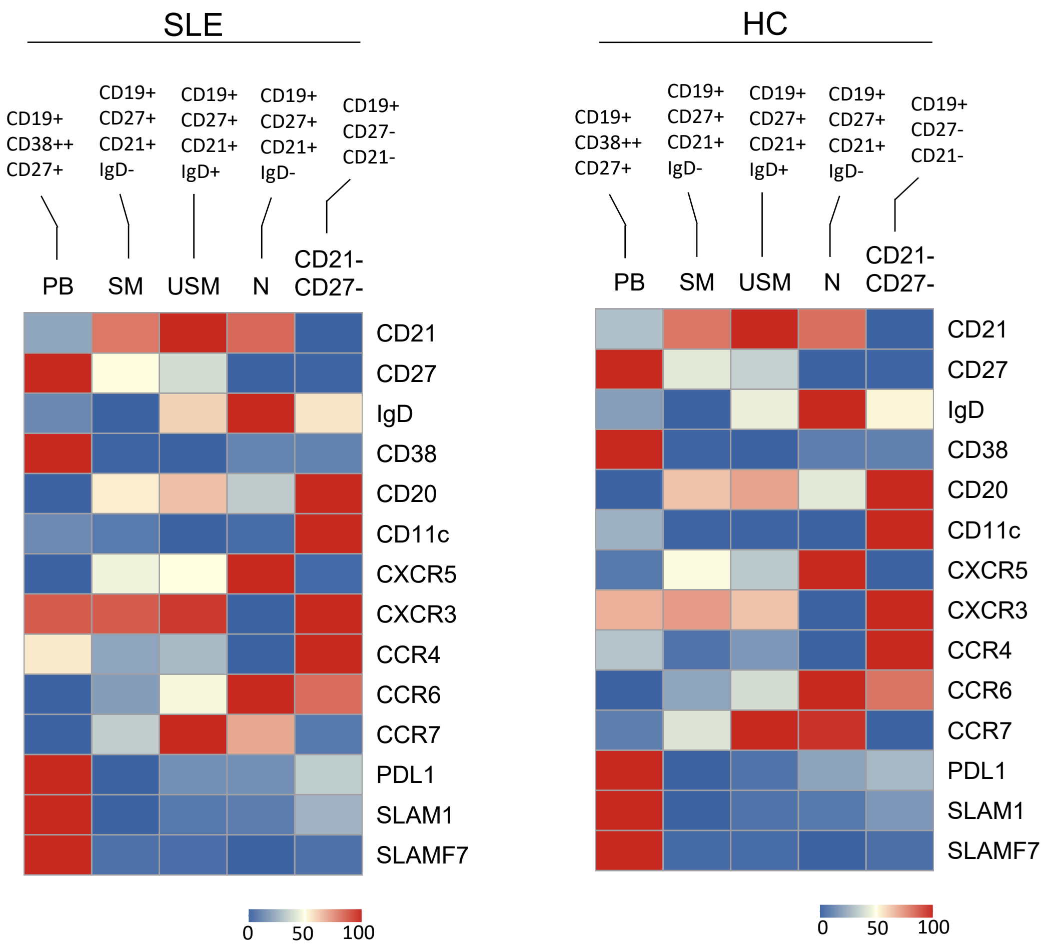

Supplementary figure 3. Heatmap showing mean levels of expression of surface markers in manually gated B cell subsets : naive (N), switched memory (SM), unswitched memory (USM), CD21^{-}CD27^{-} B cells and plasmablast (PB) in 30 systemic lupus erythematosus patients (left panel) and 30 healthy controls (right panel). B cells were analyzed by mass cytometry (cohort A). Data was normalized by rows. Figures were performed using GraphPad Prism version 8.0.0 for Windows (GraphPad Software, San Diego, California USA, www.graphpad.com).

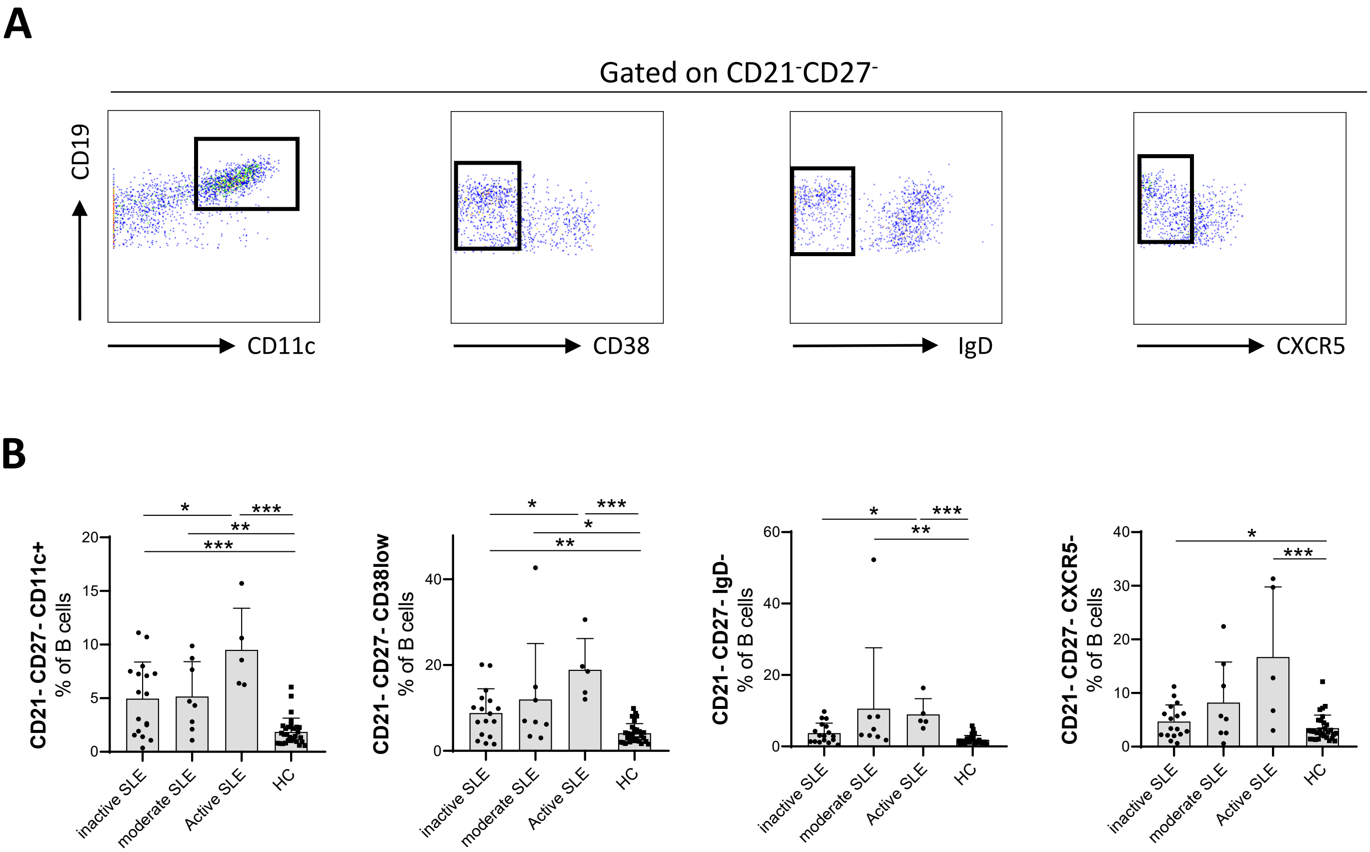

Supplementary figure 4. Using additional surface markers to define $CD21^-CD27^-$ B cells did not improve discrimination of disease activity in SLE. **(A)** Gating strategy. **(B)** Frequencies of $CD21^-CD27^-$ B cells using additional surface markers expressions (IgD-, CD38low, CD11c+, CXCR5-) in SLE patients according to disease activity and compared to HC. B cell subsets were analyzed by mass cytometry in 30 SLE patients and 30 HC (cohort A). Bar plots represents mean ± SD. Statistical analysis were performed on log-transformed data (to obtain normal distributions) using a one-way ANOVA followed by Bonferroni's correction, adjusted p value * < 0.05, ** < 0.01, *** < 0.001. Analysis and figures were performed using GraphPad Prism version 8.0.0 for Windows (GraphPad Software, San Diego, California USA, www.graphpad.com).

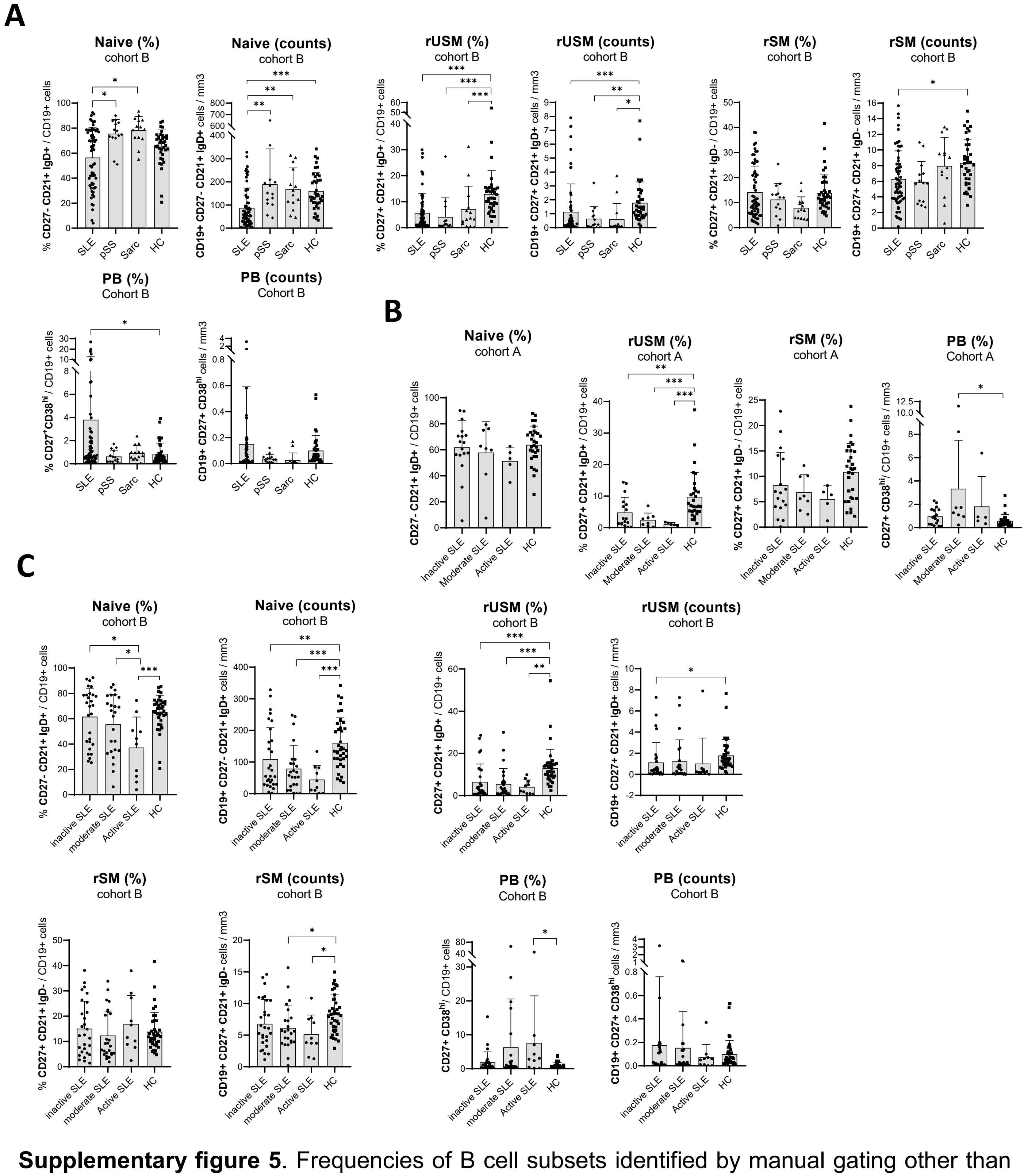

Supplementary figure 5. Frequencies of B cell subsets identified by manual gating other than CD21-CD27- B cells. **(A)** Proportions and absolute counts of B cells in SLE patients compared to controls (B cell analysis by flow cytometry of fresh blood in 63 SLE patients, 14 primary Sjögren's syndrome, 14 sarcoidosis and 39 HC = cohort B). **(B)** Proportions of B cell subsets in SLE patients according to their disease activity and compared to HC in cohort A (B cell analysis by mass cytometry in 17 inactive SLE, 8 moderate SLE, 5 active SLE and 30 HC). **(C)** Proportions and absolute counts of B cell subsets in SLE patients according to their disease activity and compared to HC in cohort B. Scatter bar plots represents mean ± SD. Statistical analysis were performed on log-transformed data (to obtain normal distributions) using a one-way ANOVA with Bonferroni correction for multiple testing, p value * < 0.05, p value ** < 0.01, p value *** < 0.001. Analysis and figures were performed using GraphPad Prism version 8.0.0 for Windows (GraphPad Software, San Diego, California USA, www.graphpad.com).
