# Supplementary material for: Measurement of circulating CD21−CD27− B lymphocytes in SLE patients is associated with disease activity independently of conventional serological biomarkers
Source: Sci Rep. 2022 Jun 2;12:9189. doi: 10.1038/s41598-022-12775-4 (PMC9163192; doi:10.1038/s41598-022-12775-4)
Supplement: Supplementary file 2 — Supplementary Tables. [file 41598_2022_12775_MOESM2_ESM.docx]

**Supplementary table 1**

**A. Characteristics of SLE patients and controls (cohort A discovery)**

|  | SLE (n= 30) | HC (n=30) |
| --- | --- | --- |
| Age, mean (SD) years | 43 (14) | 43 (14) |
| Gender, n females (%) | 25 (83) | 25 (83) |
| Ethnic background, n caucasian (%) | 27 (90) | 27 (90) |
| Disease duration since diagnosis, mean (SD) months | 86 (73) | - |
| Disease activity according to the PGA, mean score (SD) | 0.8 (0.8) | - |
| Immunosuppressive therapy*, n patients with (%) | 16 (53) | - |

**B. Characteristics of SLE patients and controls (cohort B validation)**

|  | SLE (n= 64) | pSS (n=14) | Sarc (n=14) | HC (n=39) |
| --- | --- | --- | --- | --- |
| Age, mean (SD) years | 45 (13) | 48 (18) | 48 (14) | 42 (14) |
| Gender, n females (%) | 56 (87) | 14 (100) | 2 (14) | 22 (56) |
| Ethnic background, n caucasian (%) | 44 (69) | 10 (71) | 12 (86) | - |
| Disease duration since diagnosis, mean (SD) months | 142 (175) | 50 (49) | 19 (38) | - |
| Disease activity according to the PGA, mean score (SD) | 0.9 (0.9) | 1.6 (0.5) | 1.6 (1.2) | - |
| Immunosuppressive therapy*, n patients with (%) | 33 (52) | 1 (1) | 3 (21) | - |

SLE: systemic lupus erythematosus, pSS: primary Sjögren syndrome, Sarc: sarcoidosis, HC: healthy controls, PGA: physician global assessment score, SD: standard deviation. *Defined as treatment with prednisolone or equivalent > 10 mg per day and/or any immunosuppressive drugs.

**Supplementary table 2. Antibody inventory**

| **Flow Cytometry Antibody** | **Format** | **Clone** | **Company** |
| --- | --- | --- | --- |
| CD38 | V450 | HB7 | BD bioscience |
| CD19 | APC-Cy7 | SJ25C1 (= SJ25-C1) | BD bioscience |
| IgM | FITC | MHM-88 | Biolegend |
| CD21 | PE-Cy7 | B-ly4 | BD bioscience |
| IgD | PE | IA6-2 (= δ-IA6-2) | BD bioscience |
| CD45 | PerCP | 2D1 | BD bioscience |
| CD27 | APC | M-T271 | BD bioscience |

| **Mass Cytometry Antibody** | **Format** | **Clone** | **Company** |
| --- | --- | --- | --- |
| CD45 | 89Y | HI30 | Fludigm |
| Live/Dead | 103Rh | - | Fludigm |
| CD8 | 113 In | RPA-T8 | Biolegend |
| CD4 | 115 In | RPA-T4 | Biolegend |
| CD19 | 142 Nd | HIB19 | Fludigm |
| CD352 / SLAM 6 | 143 Nd | NT-7 | Fludigm |
| CD38 | 144 Nd | HIT2 | Biolegend |
| CD127 | 145 Nd | A019D5 | Biolegend |
| IgD | 146 Nd | IA6-2 | BD bioscience |
| CD7 | 147 Sm | CD7-6B7 | Fludigm |
| CD45 | 148 Nd | HI30 | Conju-Biolegend |
| CCR4 | 149 Sm | 205410 | Fludigm |
| CD3 | 150 Nd | UCH-T1 | BD bioscience |
| PD-1 | 151 Eu | EH12.2H7 | Biolegend |
| CD21 | 152 Sm | BL13 | Fludigm |
| CD45RA | 153 Eu | HI100 | BD bioscience |
| CD84 / SLAM 5 | 154 Sm | CD84.1.21 | Fludigm |
| CD27 | 155 Gd | L128 | Fludigm |
| SLAMF 7 (CD319) | 156 Gd | 162.1 | Biolegend |
| CXCR3 | 158 Gd | 1C6/CXCR3 | BD bioscience |
| CCR7 | 159 Tb | G043H7 | Biolegend |
| CD14 | 160 Gd | M5E2 | Fludigm |
| CD150 / SLAM 1 | 161 Dy | A12(7D4) | Biolegend |
| CD11c | 162 Dy | clone 3.9 | Fludigm |
| CRTh2 (Fluidigm) | 163 Dy | BM16 | Fludigm |
| CD48 / SLAM 2 | 164 Dy | BJ40 | Biolegend |
| CD45RO | 165 Ho | UCHL1 | Fludigm |
| CD45 | 166 Er | HI30 | Conju-Biolegend |
| CXCR5 | 167 Er | RF8B2 | Biolegend |
| ICOS | 168 Er | C398.4A | Biolegend |
| CD25 | 169 Tm | 2A3 | Fludigm |
| TCR va24-Ja18 (6B11) | 170 Er | Witek | Fludigm |
| CD20 | 171Yb | 2H7 | Fludigm |
| TCRαβ | 172 Yb | IP26 | Biolegend |
| HLA-DR | 173 Yb | L243 | Fludigm |
| CD229 / SLAM 3 | 174 Yb | HLy9.1.25 | Fludigm |
| CD244 / SLAM 4 | 175Lu | C1.7 | Biolegend |
| CD56 | 176Yb | R19-760 | Fludigm |
| CD57 (CHUV) | 194 Pt | NK1 | Conju-BD bioscience |
| CD45 | 198 Pt | HI30 | Conju-Biolegend |
| CD16 | 209Bi | 3G8 | Fludigm |

**Supplementary table 3. Definitions of B cell subsets used in manual gating analysis for cohort A (analyzed by mass cytometry) and cohort B (analyzed by flow cytometry)**

|  | **CD21-**  **CD27-*** | **Naive*** | **Resting unswitched memory**  **(rUSM)*** | **Resting switched memory**  **(rSM)*** | **Plasmablasts**  **(PB)** |
| --- | --- | --- | --- | --- | --- |
| **CD19** | + | + | + | + | + |
| **CD38** |  |  |  |  | ++ |
| **CD27** | - | - | + | + | + |
| **CD21** | - | + | + | + |  |
| **IgD** |  | + | + | - |  |

* All these B cell subsets were gated within non-plasmablast B cells, mostly to prevent any overlap between resting memory cells and plasmablasts. The cells in grey were not used for the definitions of the specific B cell subsets. All cells were defined by either a positive (+) or negative (-) expression of the marker except for plasmablasts who were defined by a high expression of CD38 (++).

**Supplementary table 4**

**A. Association between CD21^-^CD27^-^ B cells and individual characteristics of SLE patients**

|  | **Cohort A (n = 30 SLE)**  (descriptive, *p value*) | **Cohort B (n = 63 SLE)**  (descriptive, *p value*) |
| --- | --- | --- |
| **Age**  in years | rs = -0.27,  95%CI = -0.58 to 0.11  *p = 0.1456* | rs = 0.11,  95%CI = -0.15 to 0.35  *p = 0.4111* |
| **Sex**  female vs male | t = 1.70,  95%CI = -0.23 to 0.05,  *p = 0.0996* | t = 1.97,  95%CI = -11.3 to 0.07,  *p = 0.0590* |
| **Ethnic background**  caucasian vs non-caucasian | t = 1.56,  95%CI = -0.08 to 0.17,  *p = 0.1301* | t = 2.25,  95%CI = - 0.02 to 0.41,  *p = 0.0284** |
| **Disease duration**  in months | rs = 0.20,  95%CI = -0.19 to 0.53,  *p = 0.2933* | rs = 0.13,  95%CI = -0.13 to 0.37,  *p = 0.3058* |
| **Treatment**  1) no treatment vs  2) anti-malarial only vs  3) any immunosuppressants | F = 0.12,  *p = 0.8865*  95%CI = -0.44 to 0.32 (1 vs 2)  95%CI = -0.46 to 0.22 (1 vs 3)  95%CI = -0.39 to 0.26 (2 vs 3) | F = 1.56,  *p = 0.2339*  95%CI = -0.30 to 0.50 (1 vs 2)  95%CI = -0.45 to 0.28 (1 vs 3)  95%CI = -0.45 to 0.07 (2 vs 3) |
| **Glucocorticoids dose**  in mg prednisone or eq. | rs = 0.05,  95%CI = -0.33 to 0.41,  *p = 0.8047* | rs = 0.28,  95%CI = -0.03 to 0.50,  *p = 0.0274** |

Age, disease duration and systemic glucocorticoid dose association with CD21-CD27- B cells frequencies were assessed by Spearman’s correlation (rs = Spearman’s coefficient). Sex and ethnic background association with CD21-CD27- B cells frequencies were evaluated using t-test (t = t statistics) on log-transformed data to obtain normal distribution. Treatment groups association with CD21-CD27- B cells frequencies were assessed by one-way ANOVA (F = F-statistic) on log-transformed data to obtain normal distribution. The 95% CI showed for treatment represent the results from Bonferroni’s multiple comparisons.

**B. Detailed treatments of SLE patients from cohort A and B**

|  | **SLE patients from cohort A** | | | **SLE patients from cohort B** | | |
| --- | --- | --- | --- | --- | --- | --- |
|  | Inactive*  (n=17) | Moderate*  (n=8) | Active* (n=5) | Inactive* (n=28) | Moderate* (n=25) | Active* (n=10) |
| **Treatments past month^#^:**  No treatment  Antimalarials only, no. (%)  Systemic GC, no. (%)  Dose, mean mg/d  Immunosuppressants, no. (%) | 4 (23)  5 (29)  7 (41)  10  6 (35) | 2 (25)  2 (25)  2 (25)  15  2 (25) | 1 (20)  1 (20)  3 (60)  12  2 (40) | 4 (14)  9 (32)  11 (39)  8  9 (32) | 3 (12)  6 (24)  15 (60)  11  13 (52) | 0  2 (20)  7 (70)  8  7 (70) |

^*^ SLE patients categorized into three groups of disease activity: inactive (SELENA-SLEDAI 0-3), moderate (SELENA-SLEDAI 4-10) and active (SELENA-SLEDAI >10).

^#^Dose of systemic glucocorticoids (GC) corresponds to prednisone or equivalent, and immunosuppressant agents included azathioprine, methotrexate, and mycophenolate mofetil used during the last 4 weeks.
